# HAX-1-Mediated Autophagy Modulation Involves N-Terminal LC3-Interacting Motifs

**DOI:** 10.3390/cells15161459

**Published:** 2026-08-14

**Authors:** Elizabeth Vafiadaki, Panagiotis Papadopoulos, Aristides G. Eliopoulos, Despina Sanoudou

**Affiliations:** 1 Center of Basic Research, Biomedical Research Foundation, Academy of Athens, 11527 Athens, Greece; lvafiadaki@bioacademy.gr (E.V.); up1068737@ac.upatras.gr (P.P.); 2 Clinical Genomics and Pharmacogenomics Unit, and Genetic and Pharmacogenetic Counseling Clinic, 4th Department of Internal Medicine, “Attikon” Hospital, Medical School, National and Kapodistrian University of Athens, 11527 Athens, Greece; 3 Department of Biology, Medical School, National and Kapodistrian University of Athens, 11527 Athens, Greece; eliopag@med.uoa.gr

**Keywords:** autophagy, HAX-1, LC3, p62, phospholamban, cardiac cell, cardioprotection

## Abstract

HS-1-associated protein X-1 (HAX-1) is a ubiquitously expressed, multifunctional protein that regulates Ca^2+^ homeostasis and cell survival in cardiac muscle. In addition to its well-established anti-apoptotic function, HAX-1 has recently been implicated in autophagy regulation. In the present study, we explored the molecular mechanisms underlying HAX-1-mediated autophagy modulation in cellular models, including cardiac-derived H9c2 myotubes. HAX-1 overexpression enhanced autophagic activity, as evidenced by decreased sequestosome-1 (p62), increased microtubule-associated protein light chain 3-II (LC3-II), enhanced LC3 puncta formation, and elevated autophagic flux. Conversely, HAX-1 knockdown attenuated autophagic activity. Mechanistically, co-immunoprecipitation assays showed that HAX-1 associates with both p62 and LC3. Bioinformatic analysis of the HAX-1 protein sequence identified two conserved LC3-interacting region (LIR) motifs within its N-terminal domain. Deletion of this LIR-containing region (HAX-1ΔLIR) reduced LC3 association, decreased autophagic activity, and impaired autophagy-dependent clearance of HAX-1 itself following autophagy induction, suggesting the importance of these motifs. At a cardiac-relevant level, HAX-1 promoted a chloroquine-sensitive reduction in protein levels of its known binding partner, phospholamban (PLN), a key regulator of sarcoplasmic reticulum (SR) Ca^2+^ cycling. These findings indicate a previously unrecognized LC3/LIR-dependent mechanism underlying HAX-1-mediated autophagy modulation and suggest a potential role for HAX-1 in SR protein proteostasis.

## 1. Introduction

HS-1-associated protein X-1 (HAX-1) was originally identified almost three decades ago as an interacting partner of HS-1, suggesting its involvement in B cell signal transduction [1]. Since then, HAX-1 has been reported to interact with numerous proteins that are associated with a wide range of physiological functions, implicating HAX-1 in multiple cellular processes [2,3]. HAX-1 is predominantly a mitochondrial protein; however, its localization to additional organelles such as the sarcoplasmic reticulum (SR) has also been reported [4,5,6]. While HAX-1 is ubiquitously expressed, its subcellular distribution and function vary among tissues depending on tissue-specific expression of its protein interactors. 

In cardiac muscle, HAX-1 interacts with phospholamban (PLN) [4], a small muscle-specific protein that acts as a key modulator of SR Ca^2+^ homeostasis and cardiac contractility through its inhibitory function on the SR Ca^2+^ transport ATPase (SERCA2a) [7]. In addition to PLN, HAX-1 binds to SERCA2a and, through these associations, is implicated in the regulation of Ca^2+^ homeostasis and contractility in the heart [8]. This was established through detailed analysis of HAX-1 mouse models, with HAX-1 overexpression in the heart being associated with depressed Ca^2+^ kinetics while downregulation of HAX-1 resulted in enhanced Ca^2+^ cycling and contractility [6,9]. As HAX-1 ablation was reported to relieve approximately half of PLN-mediated inhibition of SR Ca^2+^ transport, HAX-1 was proposed to act as a critical modulator of PLN-dependent regulation of SERCA2a activity and cardiac contractility [9]. Altogether, experimental evidence clearly illustrates the significance of HAX-1 in regulating cardiac function.

In addition to Ca^2+^ homeostasis and contractility, HAX-1 is implicated in promoting cell survival. In non-muscle (HEK293) cells, HAX-1 exhibited enhanced anti-apoptotic effects after hypoxia/reoxygenation-induced cell death upon co-expression of PLN, indicating the impact of the PLN/HAX-1 interaction in coordinating Ca^2+^ handling and cell survival [4]. In cardiomyocytes, the interaction of HAX-1 with caspase-9, an initiator caspase of apoptosis, provided direct evidence of its anti-apoptotic mode of action [10]. At the cardiac tissue level, HAX-1 was found to protect the heart against ischemia–reperfusion injury by attenuating apoptosis induced by ER stress [11]. This was associated with HAX-1 binding to the heat shock protein 90 (Hsp90), resulting in displacement of Hsp90 from inositol-requiring enzyme (IRE-1) and inhibition of IRE-1 activity to prevent ER stress–induced apoptotic responses [11].

While the anti-apoptotic effect of HAX-1 has been well documented, its role in regulation of autophagy has only recently started to emerge [12,13,14]. Autophagy, a lysosome-dependent degradative process, maintains cellular homeostasis by clearing damaged organelles and misfolded proteins, and serves as a pro-survival mechanism under stress conditions [15]. In cardiac muscle, dysregulated autophagy has been implicated in multiple pathological conditions including ischemia, cardiomyopathy, and heart failure [16,17,18,19,20]. Selective mitophagy and autophagy of ER/SR (ER-phagy) are critical for cardiac function given the central roles of these organelles in energy metabolism and Ca^2+^ cycling [21,22]. Previous work demonstrated that HAX-1 overexpression can enhance autophagy in HEK293 cells under oxidative stress and identified amino acids 127–180 as an autophagy-regulatory region [12]. More recently, HAX-1 overexpression was reported to promote chemoresistance in nasopharyngeal carcinoma by blocking autophagic flux [14]. Thus, HAX-1 has been linked to autophagy-related processes in non-cardiac cellular systems, but whether HAX-1 associates with core autophagy proteins through defined LC3-interacting mechanisms, and whether this has consequences for cardiac-relevant SR protein proteostasis, remained unclear. Moreover, although HAX-1 is an established binding partner of PLN, whether HAX-1 influences PLN abundance through an autophagy/lysosome-related pathway has not been investigated. 

We therefore hypothesized that HAX-1 promotes autophagy by engaging key components of the autophagy machinery, including p62 and LC3, through a discrete LC3-interacting region (LIR)-dependent mechanism. In the present study, we identify a conserved N-terminal LIR-containing region that supports HAX-1 association with LC3 and contributes to efficient HAX-1-mediated autophagic activity. We further show that HAX-1 promotes a chloroquine-sensitive reduction in the protein levels of its known binding partner PLN, suggesting a potential link between HAX-1-mediated autophagy and SR protein proteostasis.

## 2. Materials and Methods

### 2.1. Generation of HAX-1 Constructs

The full-length GFP-HAX-1 construct that was used in this study was generated by PCR using primers A (5′ TGGAGGGGTTCAAAGGTT 3′) and B (5′ TTGAAGGCCTCTGAGGGTTA 3′) and was subsequently cloned into the *EcoRI* and *SalI* restriction sites of the pEGFP vector (BD Biosciences Clontech, Takara Bio Saint-Germain-en-Laye, France). For the deletion construct GFP-HAX1ΔLIR, two separate PCR reactions were performed using primers A and C (5′ CAA GGTCCAGGCCCCCATATCGCTGAAGTTATCGTGGAAACGTATCCCTCC 3′) or primers D (5′ GGAGGGATACGTTTCCACGATAACTTCAGCGATATGGGGGCCTGGACCTTG 3′) and B. Primers C and D were designed to span across the LIR but do not contain the two LIRs sequence in order to attain their deletion. The resultant PCR products were mixed together and were next used as templates in a new PCR reaction using primers A and B. The PCR product was subsequently cloned into the *EcoRI* and *SalI* restriction sites of the pEGFP vector (BD Biosciences Clontech, Palo Alto, CA, USA). Authenticity of the constructs was confirmed by Sanger sequencing analysis (Eurofins Genomics, Konstanz, Germany).

The conditions used for the generation of the myc-HAX-1 and GFP-PLN or GFP-PLN-R14del constructs have been previously described [4,23]. 

### 2.2. Cell Culture and Transfections

HEK293 cells (ECACC, Salisbury, UK) were maintained in Dulbecco’s modified Eagle medium (DMEM) supplemented with 10% fetal bovine serum (FBS) (Thermo Fisher Scientific, Hillsboro, OR, USA), as previously described [4,8]. Transient transfections in HEK293 cells were performed using Lipofectamine™ 2000 (Invitrogen, Thermo Fisher Scientific) according to the manufacturer’s instructions. The myc-DDK-p62 and myc-DDK-LC3 plasmids were commercially available (OriGene Technologies, Rockville, MA, USA). 

The cardiac myoblast cell line H9c2 (ECACC) was maintained in DMEM supplemented with 10% FBS (Thermo Fisher Scientific), as previously described [23,24]. Transient transfections in H9c2 cells were performed using Lipofectamine™ 3000 (Invitrogen, Thermo Fisher Scientific), in accordance with the manufacturer’s instructions. Cell differentiation and myotube formation were induced by changing to differentiation medium (DMEM supplemented with 1% FBS and 10 nM retinoic acid). Cells were maintained in differentiation medium for 7 days, a point at which typically all cells were differentiated. Myotube formation was confirmed morphologically by the appearance of elongated, multinucleated cells (Supplementary Figure S1), consistent with our previously published characterization of this model [23,24].

### 2.3. Pharmacological Modulation of Autophagy and Autophagic Flux Measurement

Pharmacological modulation of autophagy was performed 24 h after transfection of HEK293 cells or after 7 days of differentiation of H9c2 cells. Autophagy inhibition was induced by treatment with 25 μM chloroquine (CQ) (Cell Signaling Technology, Leiden, The Netherlands) for 7 h, while application of 50 nM rapamycin (Santa Cruz Biotechnology, Heidelberg, Germany) for 2 h was used to promote autophagy. Following these treatments, the cells were harvested for Western blot or immunofluorescence analysis, as described below. Autophagic flux measurements were performed as previously described [23] using the CQ inhibition approach, which blocks autophagosome–lysosome fusion and thereby prevents lysosomal degradation of LC3-II. For each transfection condition, autophagic flux was calculated as the difference in relative LC3-II/GAPDH levels between CQ-treated and untreated cells. 

### 2.4. RNAi-Mediated Knockdown of HAX-1

Downregulation of HAX-1 protein levels was achieved by siRNA experiments. For this, HEK293 cells were transfected with HAX-1 or control scramble siRNAs (Santa Cruz Biotechnology, Heidelberg, Germany) using Lipofectamine RNAiMAX reagent in accordance with the manufacturer’s instructions (Invitrogen, Thermo Fisher Scientific). After 48 h, cells were harvested for Western blot analysis, as described below.

### 2.5. Cell Lysis and Western Blot Analysis

Following the indicated treatments described in Section 2.3, HEK293 or H9c2 cells, as appropriate for each experiment, were harvested and lysed in 1× RIPA buffer (Cell Signaling Technology) supplemented with protease inhibitors (Sigma-Aldrich, St. Louis, MO, USA). Western blot analysis was performed using LC3A/B (D3U4C) XP^®^, SQSTM1/p62 (Cell Signaling Technology), GFP (B-2) (Santa Cruz Biotechnology) and GAPDH (Sigma-Aldrich) primary antibodies and peroxidase-conjugated goat anti-rabbit (Bio-Rad, Hercules, Clearwater, FL, USA) or anti-mouse (Sigma-Aldrich) secondary antibodies. Immunoreactive bands were detected using Pierce ECL Plus reagents (Thermo Fisher Scientific) and protein quantification was performed using ImageJ 1.51n [25].

### 2.6. Immunofluorescence Analysis

Immunofluorescence studies were performed in transfected H9c2 cells after 7 days of differentiation, as previously described [23,24]. In brief, cells were fixed for 20 min at 25 °C with ice-cold methanol, washed with phosphate-buffered saline (1x PBS) and permeabilized for 30 min at 25 °C in PBS containing 0.1% Triton X-100. The cells were washed in PBS, incubated with Image-iT™ FX Signal Enhancer (Thermo Fisher Scientific) for 30 min and in blocking buffer (1x PBS, 1 mg/mL BSA, 10 mM NaN_3_) for 1 h at 25 °C. Primary antibodies for HAX-1 (BD Transduction Laboratories, Erembodegem, Belgium), SQSTM1/p62 (Cell Signaling Technology), LC3A/B (D3U4C) XP^®^ (Cell Signaling Technology) and ATP2A2/SERCA2 (Cell Signaling Technology) were diluted in blocking buffer and applied to the cells overnight at 4 °C. Following three washes with PBS, samples were counterstained for 1 h at 25 °C with Alexa Fluor anti-mouse or anti-rabbit 568 (Thermo Fisher Scientific) secondary antibodies diluted in blocking buffer. In untransfected H9c2 cells, Alexa Fluor anti-mouse or anti-rabbit 488 and Alexa Fluor anti-rabbit or anti-mouse 568 (Thermo Fisher Scientific) secondary antibodies were used. After three washes with PBS, the samples were mounted with Fluoroshield mounting medium supplemented with DAPI (Abcam, Cambridge, UK).

Mitochondrial labeling by MitoTracker staining was performed as previously described [4]. In brief, cultured cells were incubated for 20 min at 37 °C with MitoTracker CMXRos Red dye (Thermo Fisher Scientific) and, after three washes with PBS, cells were fixed and permeabilized, as described above.

Image acquisition was performed on a Leica confocal laser scanning microscope (Leica TCS SP5 II on a DM 6000 CFS Upright Microscope, Leica Microsystems, Wetzlar, Germany) using the LAS-AF software program (Leica Application Suite X).

LC3 puncta were quantified using ImageJ by counting discrete LC3-positive punctate structures per GFP-positive cell from confocal images acquired under identical settings. The same thresholding criteria were applied across experimental groups. For quantitative imaging analyses, cells were quantified across independent experiments, and statistical comparisons were performed using independent experiments as the biological replicate unit.

### 2.7. Immunoprecipitation Assays in Transiently Transfected HEK293 Cells

Immunoprecipitation experiments were performed in transiently transfected HEK293 cells as previously described [24]. Briefly, 24 h after transfection, cells were harvested and lysed in 1× RIPA buffer (Cell Signaling Technology) supplemented with protease inhibitors. Protein extracts were incubated overnight on a rotary wheel at 4 °C with myc antibody conjugated to agarose beads (Santa Cruz Biotechnology). Immunoprecipitates were collected at 2000 rpm for 5 min, washed in PBS and analyzed by Western blot analysis using myc (9E10) (Santa Cruz Biotechnology) or GFP (Santa Cruz Biotechnology) antibodies. The VeriBlot for IP Detection Reagent (HRP) (AbCam, Cambridge, UK) was used instead of a secondary antibody in order to allow for the detection of the co-immunoprecipitated proteins without interference from denatured heavy and light antibody chains. 

### 2.8. Statistical Analysis

Statistical analyses were performed in GraphPad Prism 8.0.2 software (GraphPad Software, Inc., La Jolla, CA, USA). Comparisons among multiple experimental groups were performed using one-way analysis of variance (ANOVA) followed by post hoc Tukey’s test for pairwise comparisons. For comparisons between two experimental groups, data distribution was assessed by the Shapiro–Wilk test before applying a two-tailed Student’s *t*-test. A *p*-value of <0.05 was considered to be statistically significant. The data are presented as mean ± standard error of the mean (SEM).

## 3. Results

### 3.1. HAX-1 Overexpression Promotes Autophagy

**Figure 1:**
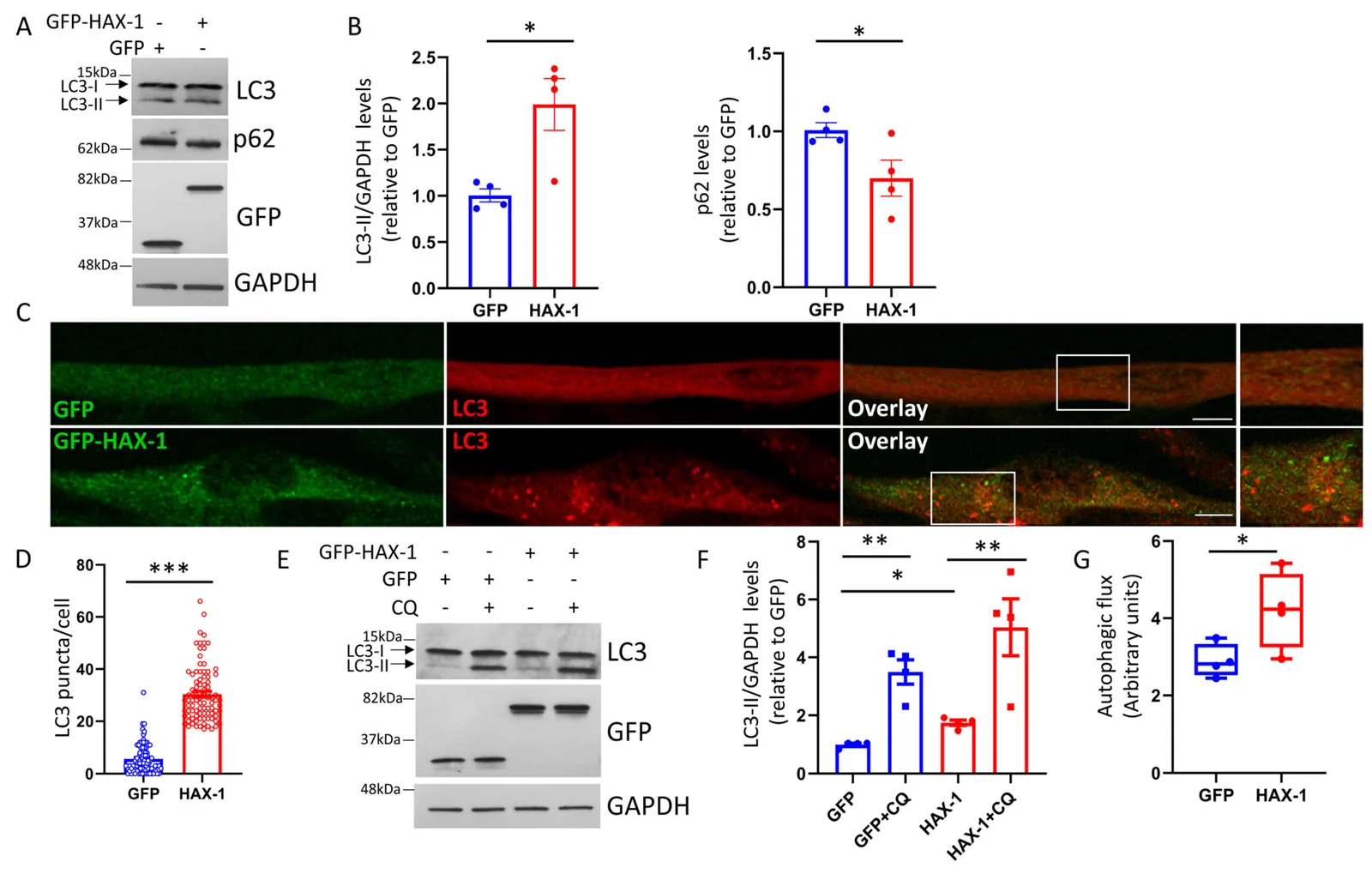
HAX-1 overexpression promotes autophagy. (**A**) Representative Western blot analysis of HEK293 cells transfected with GFP-HAX-1 or GFP control, showing LC3-I, LC3-II, p62, GFP, GFP-HAX-1 and GAPDH protein levels. Molecular weight markers are indicated. (**B**) Quantification of p62 and LC3-II levels normalized to GAPDH showed decreased p62 and increased LC3-II in GFP-HAX-1-transfected cells compared with GFP-transfected controls, consistent with increased autophagic activity (n = 4 independent experiments, * *p* < 0.05 vs. GFP; two-tailed Student’s *t*-test). (**C**) Representative immunofluorescence images of differentiated H9c2 cardiac myotubes transfected with GFP-HAX-1 or GFP control. GFP or GFP-HAX-1 is shown in green and endogenous LC3 in red. GFP-HAX-1-expressing cells displayed a punctate LC3 pattern, consistent with increased autophagosome formation, whereas GFP-expressing control cells showed predominantly diffuse LC3 staining. Zoom-in view of white box area is shown. Scale bar, 10 μm. (**D**) Quantification of LC3-positive puncta per GFP-positive cell confirmed a significant increase in LC3 puncta following GFP-HAX-1 expression. A total of 85–90 cells per condition were analyzed across 4 independent experiments; statistical analysis was performed using experiment-level values. *** *p* < 0.0001 vs. GFP; two-tailed Student’s *t*-test. (**E**) Representative Western blot analysis of LC3-I and LC3-II in GFP- or GFP-HAX-1-transfected HEK293 cells under basal conditions and following chloroquine (CQ) treatment. GAPDH was used as a loading control. (**F**) Quantification of LC3-II levels normalized to GAPDH showed increased LC3-II accumulation after CQ treatment, with a greater increase in GFP-HAX-1-transfected cells than in GFP-transfected controls (n = 4 independent experiments; * *p* < 0.05, ** *p* < 0.001; one-way ANOVA followed by Tukey’s post hoc test). (**G**) Autophagic flux was calculated for each transfection condition as the difference in relative LC3-II/GAPDH levels between CQ-treated and untreated cells [Δ relative LC3-II/GAPDH = LC3-II/GAPDH_CQ − LC3-II/GAPDH_untreated] and is expressed as arbitrary units. HAX-1 overexpression significantly enhanced autophagic flux compared with the GFP control (n = 4 independent experiments; * *p* < 0.05; two-tailed Student’s *t*-test). The data are expressed as the mean ± SEM.

To establish the experimental basis for subsequent mechanistic studies, we examined the expression of established autophagic marker proteins p62 and LC3 in HEK293 cells transfected with GFP-HAX-1 or GFP control. HAX-1 overexpression resulted in a statistically significant decrease in p62 and an increase in LC3-II levels, compared with GFP-transfected cells (Figure 1A,B). Because p62 is selectively degraded by autophagy and LC3-II represents the lipidated form of LC3 that marks autophagosome membranes, the concomitant changes in both markers are indicative of autophagy activation [15,26].

We next examined whether this biochemical phenotype was reflected at the cellular level in differentiated H9c2 cardiac myotubes. GFP-HAX-1-expressing cells displayed a more pronounced punctate LC3 distribution, consistent with increased autophagosome formation, whereas GFP-expressing control cells showed a predominantly diffuse LC3 pattern (Figure 1C). Quantification of LC3-positive puncta in GFP-positive cells confirmed a significant increase in LC3 puncta per cell following HAX-1 overexpression (Figure 1D).

Given the dynamic nature of autophagy, we next assessed autophagic flux to determine whether the observed LC3-II accumulation reflected increased autophagosome formation and turnover rather than impaired autophagosome clearance [15,26,27]. Cells were treated with chloroquine (CQ), which prevents autophagosome–lysosome fusion and thereby prevents lysosomal degradation of LC3-II [23,28]. CQ treatment increased LC3-II levels in both GFP and GFP-HAX-1 transfected cells; however, the magnitude of LC3-II accumulation was significantly greater in GFP-HAX-1 cells (Figure 1E,F). Direct calculation of autophagic flux, defined as the difference in relative LC3-II/GAPDH levels between CQ-treated and untreated cells, confirmed enhanced autophagic flux in GFP-HAX-1 cells (Figure 1G). These findings were further supported at the cellular level by a significantly greater CQ-induced accumulation of LC3 puncta in GFP-HAX-1-expressing H9c2 cells compared with GFP controls (Supplementary Figure S2). Collectively, these results demonstrate that HAX-1 overexpression enhances autophagic flux.

**Figure 2:**
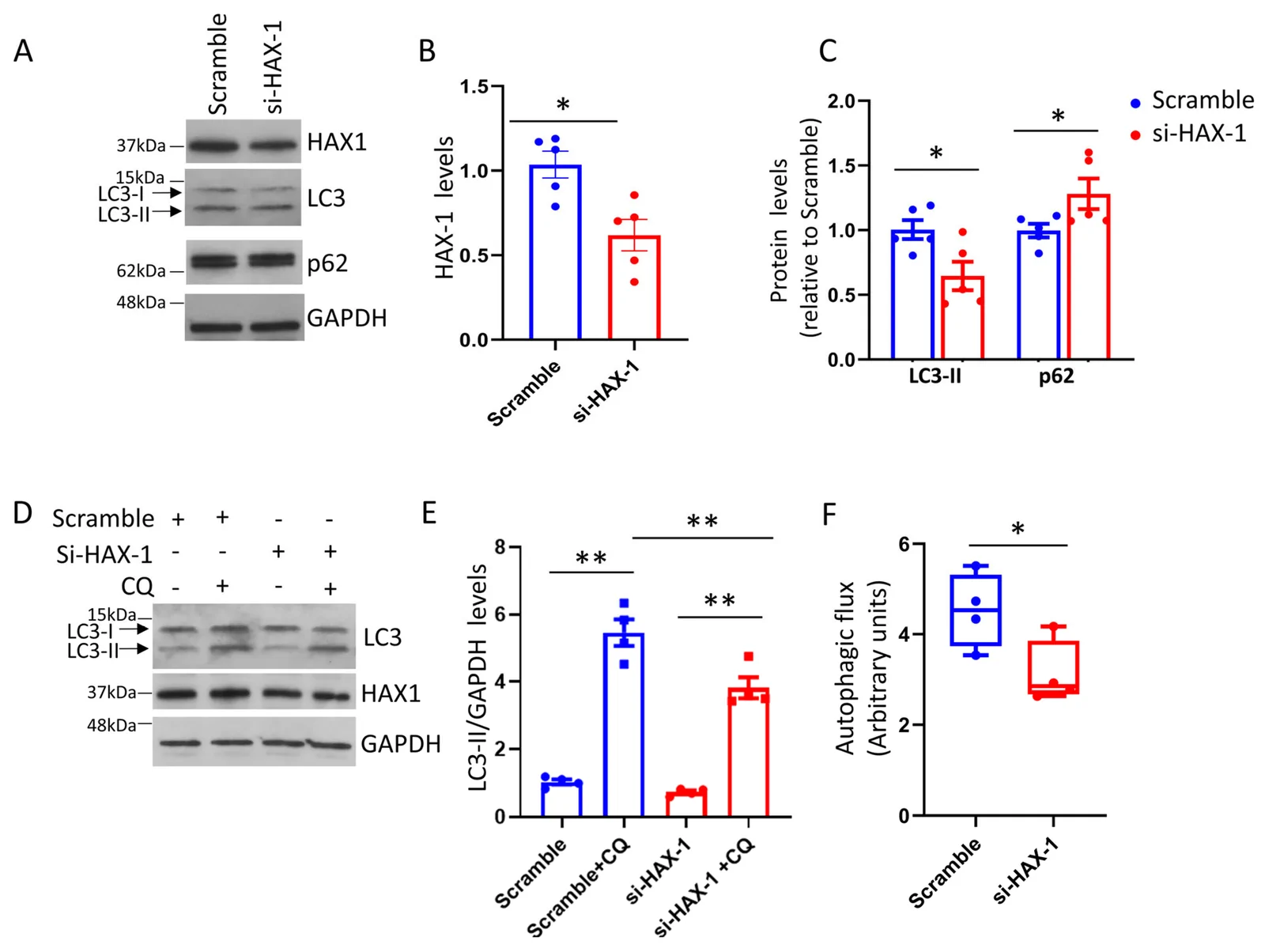
HAX-1 down-regulation depresses autophagy activation. (**A**) Representative Western blot analysis of HEK293 cells transfected with scramble control siRNA or si-HAX-1, showing HAX-1, LC3-I, LC3-II, p62 and GAPDH protein levels. (**B**) Quantification of HAX-1 levels normalized to GAPDH confirmed efficient HAX-1 downregulation following si-HAX-1 transfection (n = 5 independent experiments; * *p* < 0.05 vs. scramble; two-tailed Student’s *t*-test). (**C**) Quantification of LC3-II and p62 levels normalized to GAPDH showed decreased LC3-II and increased p62 in si-HAX-1-transfected cells compared with scramble controls, consistent with reduced autophagic activity (n = 5 independent experiments; * *p* < 0.05 vs. scramble; two-tailed Student’s *t*-test). (**D**) Representative Western blot analysis of LC3-I and LC3-II in scramble or si-HAX-1-transfected HEK293 cells under basal conditions and following chloroquine (CQ) treatment. GAPDH was used as a loading control. (**E**) Quantification of LC3-II levels normalized to GAPDH showed altered CQ-induced LC3-II accumulation in si-HAX-1-transfected cells compared with scramble controls (n = 5 independent experiments; ** *p* < 0.001; one-way ANOVA followed by Tukey’s post hoc test). (**F**) Autophagic flux was calculated for each siRNA condition as the difference in relative LC3-II/GAPDH levels between CQ-treated and untreated cells [Δ relative LC3-II/GAPDH = LC3-II/GAPDH_CQ − LC3-II/GAPDH_untreated] and is expressed as arbitrary units. HAX-1 downregulation significantly reduced autophagic flux compared with the scramble control (n = 4 independent experiments; * *p* < 0.05 vs. scramble; two-tailed Student’s *t*-test). The data are expressed as mean ± SEM.

To complement these gain-of-function findings, we performed siRNA-mediated knockdown of HAX-1 in HEK293 cells. Reduction in HAX-1 protein levels produced the inverse autophagic phenotype with decreased LC3-II, increased p62 levels, and reduced autophagic flux compared with scramble siRNA controls (Figure 2A–F), consistent with suppressed autophagy. Together, these gain- and loss-of-function data support a positive regulatory role for HAX-1 in autophagic activity.

### 3.2. HAX-1 Associates with p62 and LC3 Proteins

**Figure 3:**
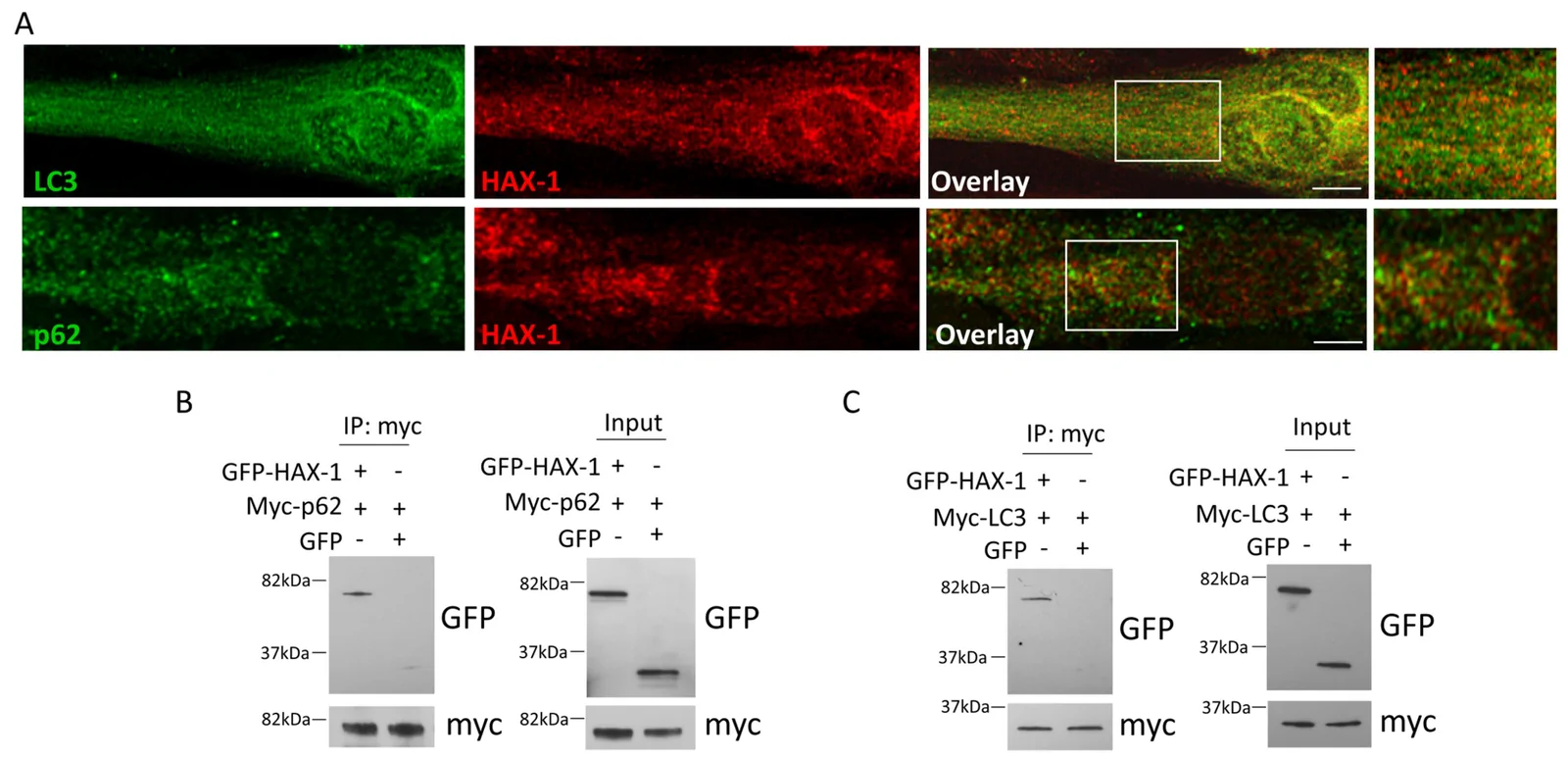
HAX-1 associates with autophagy proteins p62 and LC3. (**A**) Immunofluorescence analysis of endogenously expressed proteins in H9c2 myotubes revealed partial colocalization of HAX-1 with p62 and LC3, being mostly evident around puncta sites. Zoom-in view of white box area is shown on the right. Scale bar, 10 μm. (**B**) Immunoprecipitation assays in HEK293 cells co-transfected with GFP-HAX-1 and myc-p62 (left panel) or (**C**) myc-LC3 (left panel) determined the interaction of HAX-1 with these two autophagy-related proteins. Input (right panels) represents an aliquot (10%) of the lysates used in the immunoprecipitation assays.

Having established a positive regulatory role for HAX-1 in autophagic activity, we next investigated the molecular mechanisms mediating this effect. Towards this, we evaluated the subcellular distribution of endogenously expressed HAX-1 and autophagy marker proteins p62 or LC3 in differentiated H9c2 cardiac myotubes. Immunofluorescence analysis revealed partial colocalization of HAX-1 with both p62 and LC3, with most pronounced overlap at punctate structures (Figure 3A), consistent with recruitment of a fraction of HAX-1 to p62/LC3-positive autophagy-related structures. This finding is further supported by a recent proteomics study identifying p62 as a HAX-1-interacting protein [29]. 

To examine the potential interaction of HAX-1 with p62 and LC3 biochemically, we performed co-immunoprecipitation assays in HEK293 cells co-transfected with GFP-HAX-1 and either myc-p62 or myc-LC3. GFP-HAX-1 was co-immunoprecipitated with myc-p62, whereas GFP alone was not (Figure 3B), supporting an association between HAX-1 and p62. Similarly, GFP-HAX-1 was co-immunoprecipitated with myc-LC3, whereas GFP control showed no binding (Figure 3C). The relatively low levels of co-immunoprecipitated GFP-HAX-1 are consistent with the partial colocalization observed by immunofluorescence and may reflect that only a subfraction of cellular HAX-1 engages p62- or LC3-containing complexes under these conditions. Collectively, these data demonstrate that HAX-1 associates with both p62 and LC3 in a cellular context. 

### 3.3. The N-Terminal Region of HAX-1 Contains Conserved LIR Motifs Required for Optimal LC3 Association and Autophagy Activation

**Figure 4:**
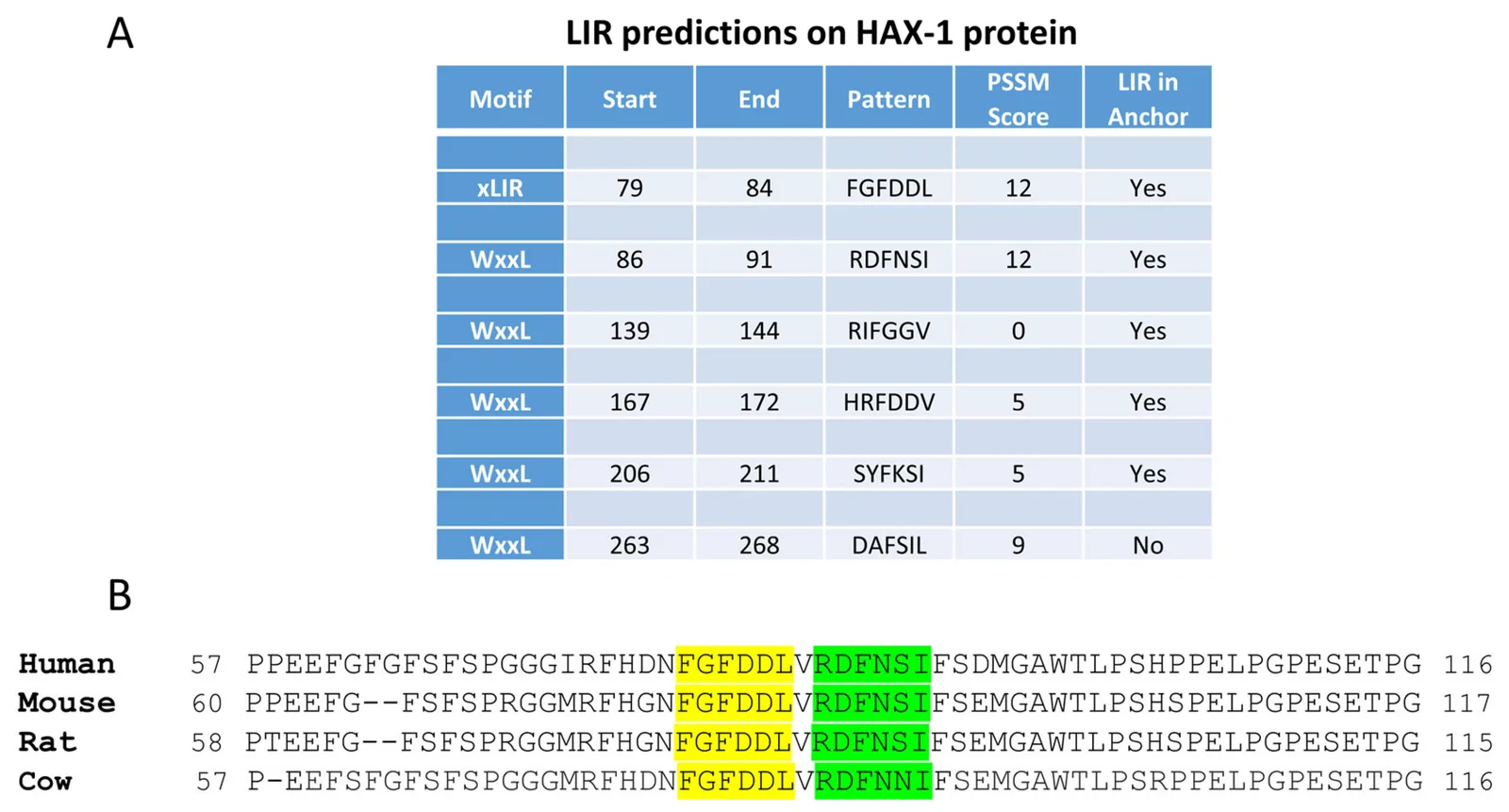
HAX-1 protein sequence is predicted to contain LIR motifs that are conserved among species. (**A**) Bioinformatic analysis of the HAX-1 sequence by the iLIR Autophagy Database predicted the existence of several putative LIR motifs within HAX-1. For each predicted motif, the start and end amino acid positions on the HAX-1 protein, as well as the corresponding sequence pattern, are shown. A High positive Position Specific Scoring Matrix (PSSM) score, along with a positive prediction by the ANCHOR dataset (LIR in anchor), are strong indicators of putative LIRs. (**B**) Alignment of mammalian HAX-1 amino acid residues around the two LIRs (LIR1 highlighted in yellow and LIR2 in green, respectively) revealed a high degree of conservation among species, suggesting their putative functional significance.

The LC3-interacting region (LIR) is a short amino acid motif that is found in several selective autophagy receptors and adaptor proteins and is known to be required for binding to LC3 during selective autophagy [30,31,32]. Having identified the HAX-1/LC3 association, we searched the HAX-1 sequence for putative LIR motifs using the iLIR Autophagy Database (https://ilir.warwick.ac.uk/index.php (accessed 11 January 2024). Multiple candidate LIR motifs were identified; among these, two motifs located at amino acid positions 79–84 (LIR1: FGFDDL) and 86–91 (LIR2: RDFNSI) exhibited the highest prediction scores (Position Specific Scoring Matrix [PSSM] score 12 for both motifs) and were positively identified by the ANCHOR dataset (Figure 4A). Importantly, multiple species sequence alignment of the HAX-1 region encompassing these two motifs demonstrated that these two motifs are conserved across mammalian species (Figure 4B), supporting their potential functional relevance. Notably, these predicted LIR motifs lie outside the previously reported HAX-1 amino acid 127–180 autophagy-regulatory region, indicating that they may define a distinct LC3-interacting mechanism.

**Figure 5:**
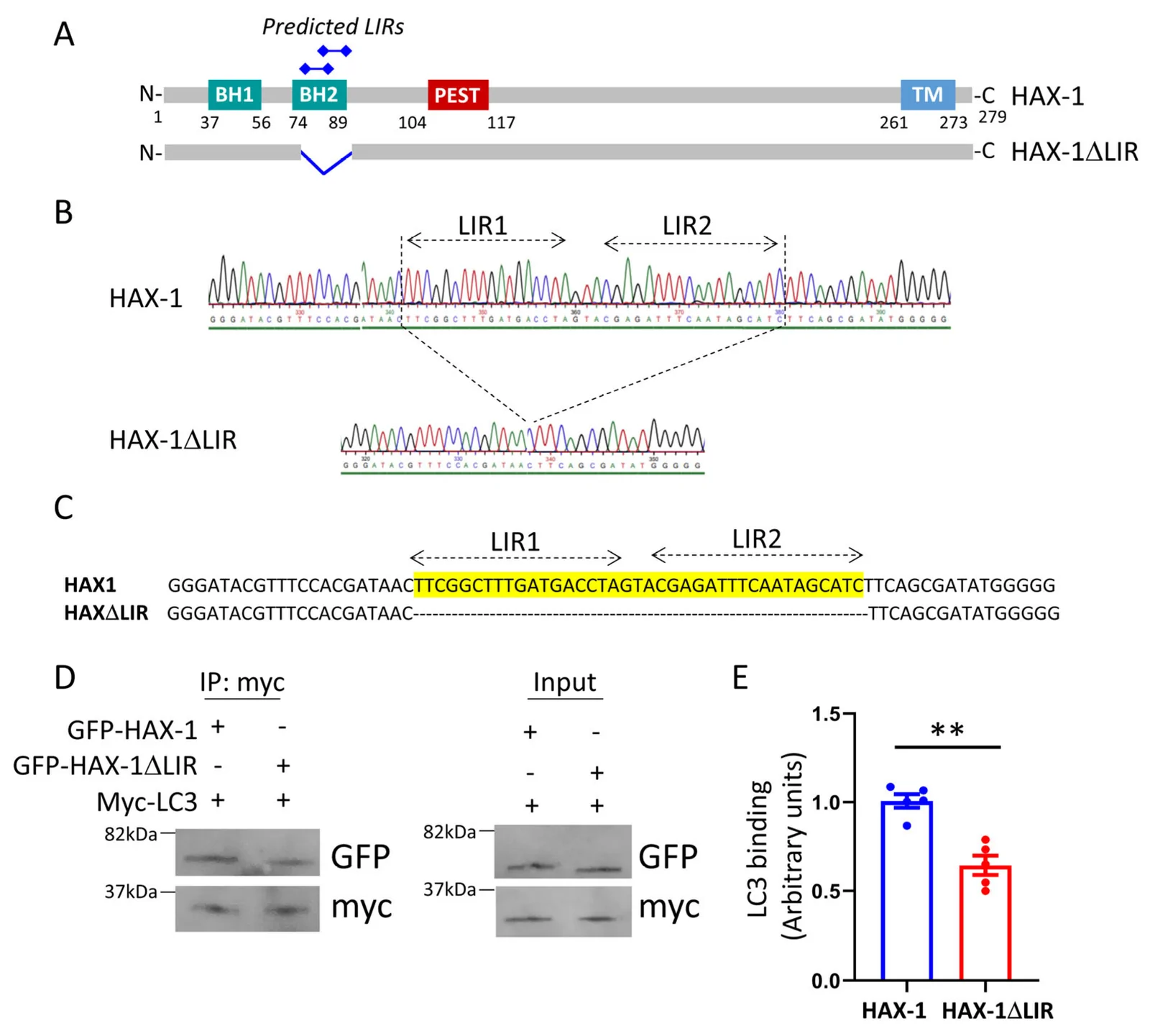
The LIR-containing N-terminal region of HAX-1 is required for efficient LC3 association. (**A**) Schematic representation of full-length HAX-1 and HAX-1ΔLIR proteins. The Bcl-2 homology domains 1 and 2 (BH1 and BH2), PEST sequence and transmembrane domain (TM) are indicated, with the location of the two predicted LIR motifs being depicted by the blue lines. (**B**) Electropherogram and (**C**) alignment of the sequence encompassing the predicted LIR domains (highlighted in yellow) confirm their deletion in the GFP-HAX-1ΔLIR construct. (**D**) Immunoprecipitation assays in HEK293 cells co-transfected with myc-LC3 and GFP-HAX-1 or GFP- HAX-1ΔLIR showed reduced co-immunoprecipitation of GFP-HAX-1ΔLIR with myc-LC3 (left panel). Input (right panel) represents an aliquot (10%) of the lysates used in the immunoprecipitation assays. (**E**) Quantification of co-immunoprecipitated GFP-HAX-1 or GFP-HAX-1ΔLIR signal revealed significantly reduced association of HAX-1ΔLIR with LC3 compared with full-length HAX-1 (n = 5 experiments, ** *p* < 0.01 vs. GFP-HAX-1; two-tailed Student’s *t*-test).

To test the functional role of these LIR motifs in HAX-1’s association with LC3, we generated a deletion construct (GFP-HAX-1ΔLIR) lacking amino acids 79–91 (Figure 5A). Deletion was confirmed by Sanger sequencing and sequence alignment to the full-length HAX-1 construct (Figure 5B,C). We next performed co-immunoprecipitation assays to assess whether this LIR-containing region contributes to HAX-1 association with LC3. These experiments revealed significantly reduced co-immunoprecipitation of GFP-HAX-1ΔLIR to myc-LC3 compared with full-length GFP-HAX-1 (Figure 5D,E), supporting the conclusion that the N-terminal LIR-containing region contributes to efficient HAX-1 association with LC3.

**Figure 6:**
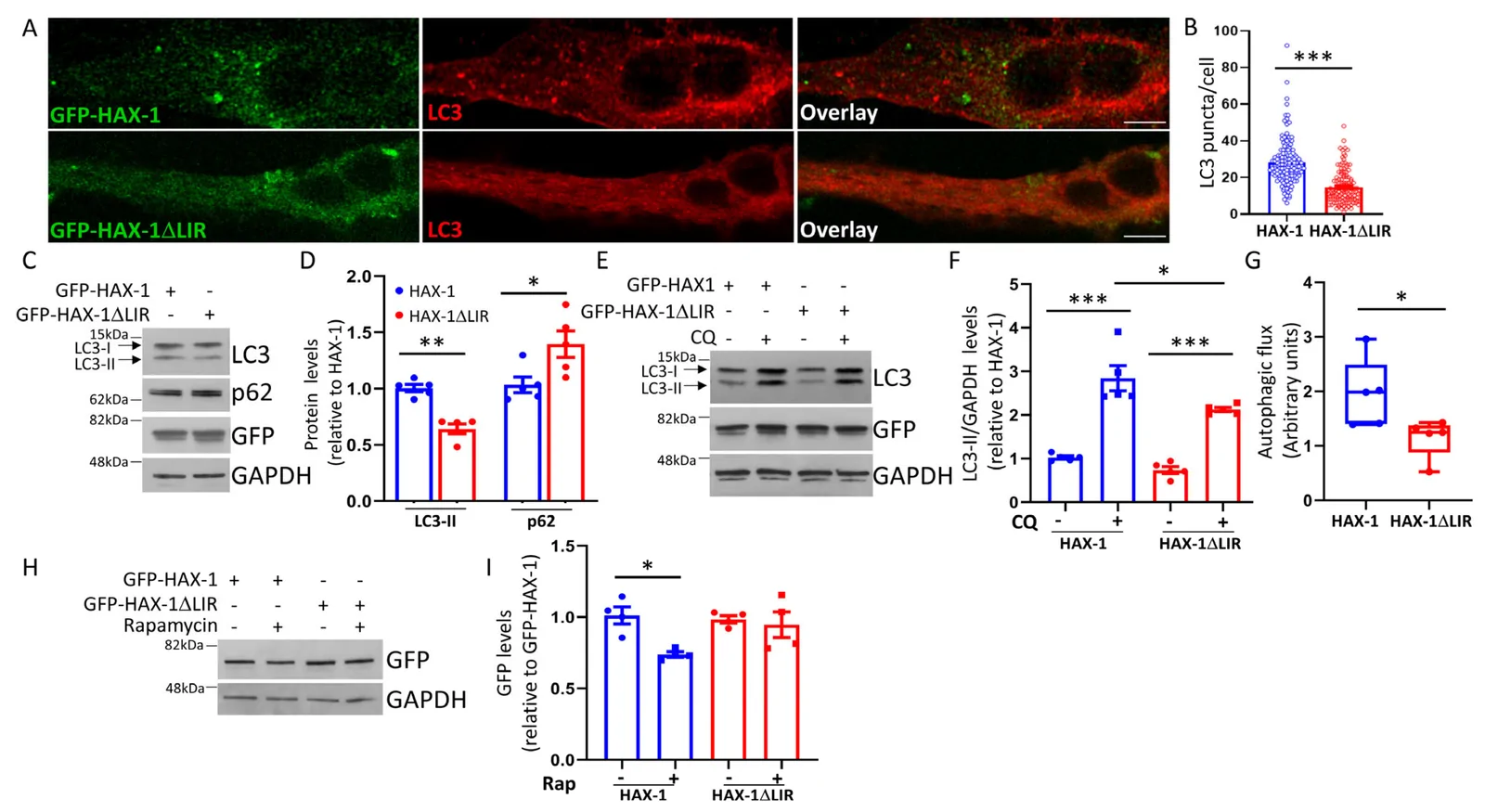
The N-terminal LIR-containing region of HAX-1 is required for efficient autophagy activation. (**A**) Representative immunofluorescence analysis of differentiated H9c2 cells transfected with GFP-HAX-1 or GFP-HAX-1ΔLIR and stained for LC3. GFP-HAX-1ΔLIR-expressing cells displayed reduced punctate LC3 distribution compared with GFP-HAX-1-expressing cells. Scale bar, 10 μm. (**B**) Quantification of LC3-positive puncta per GFP-positive cell showed a significant decrease in GFP-HAX-1ΔLIR-expressing cells compared with GFP-HAX-1-expressing cells. A total of 126–146 cells per condition were analyzed across 3 independent experiments; statistical analysis was performed using experiment-level values. *** *p* < 0.0001 vs. GFP-HAX-1; two-tailed Student’s *t*-test. (**C**) Representative Western blot analysis of HEK293 cells transfected with GFP-HAX-1 or GFP-HAX-1ΔLIR, showing LC3-I, LC3-II, p62, GFP-HAX-1, GFP-HAX-1ΔLIR and GAPDH protein levels under basal conditions. (**D**) Quantification of LC3-II and p62 levels normalized to GAPDH revealed reduced LC3-II and increased p62 in GFP-HAX-1ΔLIR-expressing cells compared with GFP-HAX-1-expressing cells, consistent with reduced autophagic activity (n = 5 independent experiments; * *p* < 0.05, ** *p* < 0.01 vs. GFP-HAX-1; one-way ANOVA followed by Tukey’s post hoc test). (**E**) Representative Western blot analysis of LC3-I and LC3-II in GFP-HAX-1- or GFP-HAX-1ΔLIR-transfected HEK293 cells under basal conditions and following chloroquine (CQ) treatment. GAPDH was used as a loading control. (**F**) Quantification of LC3-II levels normalized to GAPDH showed reduced CQ-induced LC3-II accumulation in GFP-HAX-1ΔLIR-expressing cells compared with GFP-HAX-1-expressing cells (n = 5 independent experiments; * *p* < 0.05, *** *p* < 0.0001; one-way ANOVA followed by Tukey’s post hoc test). (**G**) Autophagic flux was calculated for each transfection condition as the difference in relative LC3-II/GAPDH levels between CQ-treated and untreated cells [Δ relative LC3-II/GAPDH = LC3-II/GAPDH_CQ − LC3-II/GAPDH_untreated] and is expressed as arbitrary units. GFP-HAX-1ΔLIR-expressing cells showed significantly reduced autophagic flux compared with GFP-HAX-1-expressing cells (n = 5 independent experiments; * *p* < 0.05 vs. GFP-HAX-1; two-tailed Student’s *t*-test). (**H**) Representative Western blot analysis of GFP-HAX-1 and GFP-HAX-1ΔLIR protein levels in transfected HEK293 cells under basal conditions and following autophagy induction by rapamycin (Rap) treatment. GAPDH was used as a loading control. (**I**) Quantification showed a significant reduction in GFP-HAX-1 protein levels after rapamycin treatment, whereas GFP-HAX-1ΔLIR protein levels were not significantly altered. These findings indicate that the LIR-containing region contributes to autophagy-dependent clearance of HAX-1 (n = 4 independent experiments; * *p* < 0.05; two-tailed Student’s *t*-test). The data are expressed as the mean ± SEM.

We next evaluated the functional consequences of LIR deletion for autophagic activity. Immunofluorescence analysis in H9c2 cardiac myotubes revealed significantly fewer LC3 puncta in GFP-HAX-1ΔLIR than GFP-HAX-1-expressing cells (Figure 6A,B). Western blot analysis showed reduced LC3-II and elevated p62 in GFP-HAX-1ΔLIR-expressing cells compared with GFP-HAX-1 controls (Figure 6C,D), and autophagic flux was significantly reduced in HAX-1ΔLIR-expressing cells (Figure 6E–G). Furthermore, while induction of autophagy by rapamycin treatment reduced GFP-HAX-1 protein levels, GFP-HAX-1ΔLIR levels remained unaffected (Figure 6H,I). Collectively, these findings support a role for the N-terminal LIR-containing region of HAX-1 in LC3 association and HAX-1-mediated autophagy activation.

### 3.4. HAX-1 Is Implicated in Autophagic Degradation of PLN and Regulates Its Protein Levels

**Figure 7:**
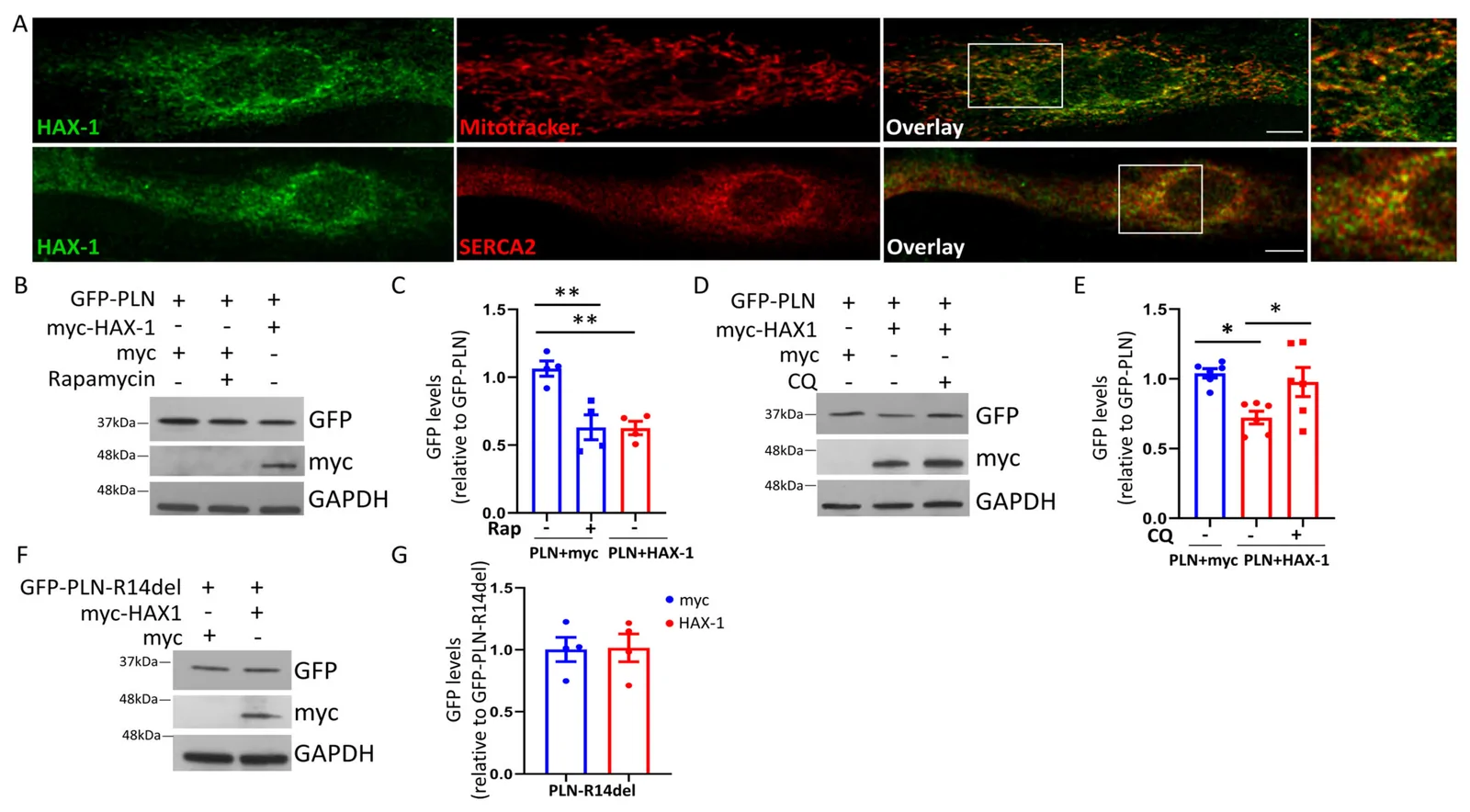
HAX-1 promotes autophagic/lysosomal regulation of the SR protein PLN. (**A**) Representative immunofluorescence analysis of endogenously expressed proteins in differentiated H9c2 myotubes showing partial colocalization of HAX-1 with the mitochondrial marker MitoTracker and the SR marker SERCA2. Zoom-in view of white box area is shown on the right. Scale bar, 10 μm. (**B**) Representative immunoblot analysis of GFP-PLN levels in HEK293 cells transfected with GFP-PLN under three conditions: co-transfection with myc control, co-transfection with myc control followed by rapamycin (Rap) treatment, or co-transfection with myc-HAX-1. GAPDH was used as a loading control. (**C**) Quantification of GFP-PLN levels showed that rapamycin significantly reduced GFP-PLN abundance. Co-expression of myc-HAX-1 similarly reduced GFP-PLN levels, supporting a role for HAX-1 in promoting PLN turnover through an autophagy-related pathway (n = 4 independent experiments; ** *p* < 0.001; one-way ANOVA followed by Tukey’s post hoc test). (**D**) Representative Western blot analysis of GFP-PLN levels in HEK293 cells co-transfected with GFP-PLN and myc-HAX-1 in the absence or presence of chloroquine (CQ). GAPDH was used as a loading control. (**E**) Quantification showed that CQ treatment restored GFP-PLN levels in the presence of HAX-1, indicating that the HAX-1-mediated reduction in PLN is CQ-sensitive and depends on the autophagic/lysosomal pathway (n = 6 independent experiments; * *p* < 0.05; one-way ANOVA followed by Tukey’s post hoc test). (**F**) Representative Western blot analysis of GFP-PLN-R14del levels in HEK293 cells co-transfected with myc or myc-HAX-1. GAPDH was used as a loading control. (**G**) Quantification of GFP-PLN-R14del levels determined no change in its expression levels by myc-HAX-1 co-expression, potentially due to underlying autophagic defects associated with PLN-R14del (n = 4 independent experiments). The data are expressed as mean ± SEM.

In cardiac muscle, HAX-1 is known to exhibit a dual localization pattern, being present in both mitochondria and SR [5,6]. This dual subcellular distribution of HAX-1 was also observed in differentiated H9c2 cardiac myotubes, with endogenously expressed HAX-1 partially co-localizing with markers for both mitochondria and SR (Figure 7A). 

The SR protein PLN has previously been reported to undergo autophagic degradation in cardiomyocytes [33]. Given that PLN is a known HAX-1-binding partner and that HAX-1 promotes autophagy, we examined whether HAX-1 modulates PLN protein levels through an autophagy-related pathway. In GFP-PLN-transfected HEK293 cells, rapamycin treatment significantly reduced PLN protein levels (Figure 7B,C), consistent with prior observations [33]. Interestingly, co-expression of myc-HAX-1 reduced PLN levels to an extent comparable to rapamycin treatment alone, even in the absence of rapamycin (Figure 7B,C). HAX-1 co-expression also reduced GFP-PLN levels in transfected H9c2 cells, supporting the relevance of this effect in a cardiac-derived cellular model (Supplementary Figure S3). 

To confirm that this HAX-1-driven reduction in PLN levels is lysosome-dependent, we treated PLN/HAX-1 co-expressing cells with CQ. Lysosomal inhibition by CQ restored PLN levels in the presence of HAX-1 (Figure 7D,E), supporting the conclusion that HAX-1 promotes PLN reduction through an autophagic/lysosomal pathway. In support of this, the presence of HAX-1 failed to promote reduction in the protein levels of the disease-associated human PLN variant (c.40_42delAGA, p.ArgR14del) PLN-R14del (Figure 7F,G). This is not due to disruption of PLN-R14del binding to HAX-1 [24] but instead may be attributed to PLN-R14del-mediated autophagy defects [23,34], occurring at the late stages of autophagosome–lysosome fusion that therefore hinder protein clearance [23]. These findings implicate HAX-1 in the regulation of SR protein proteostasis through autophagy, with potential consequences for SR Ca^2+^ homeostasis and cardiac function.

## 4. Discussion

The present study identifies a previously unrecognized LC3/LIR-dependent mechanism contributing to autophagy regulation by HAX-1. Previous work demonstrated that HAX-1 can promote autophagy in HEK293 cells under oxidative stress and identified amino acids 127–180 as an autophagy-regulatory region [12]. In addition, HAX-1 has recently been implicated in autophagy-related chemoresistance in nasopharyngeal carcinoma, where HAX-1 overexpression was reported to block autophagic flux [14]. Our findings extend these observations by showing that HAX-1 associates with the autophagy proteins p62 and LC3, and by mapping LC3 association to a predicted N-terminal LIR-containing region that lies outside the previously described amino acid 127–180 HAX-1 region. Deletion of the LIR-containing region reduces LC3 association, decreases autophagic activity and impacts autophagy-dependent clearance of HAX-1 itself. At the functional cell-based level, our findings indicate that HAX-1 promotes CQ-sensitive, lysosome-dependent reduction in its SR binding partner PLN. Because regulation of PLN levels by autophagy has previously been demonstrated in cardiomyocytes [33], our data suggest a potential link between HAX-1-mediated autophagy and PLN/SR protein proteostasis, which warrants further validation in primary cardiomyocytes and in vivo cardiac models.

The identification of two functional LIR motifs in HAX-1 places it within a growing group of pro-survival and Bcl-2-related proteins that regulate both apoptosis and autophagy through LIR-dependent LC3 interactions, including Bcl2L13, BNIP3, and NIX [35,36,37]. Bioinformatic analysis also predicts putative LIR motifs in Bcl-2 and BclxL (Supplementary Figure S4), further supporting this mechanistic parallel. Like these proteins, HAX-1 may coordinate pro-survival responses by modulating both apoptotic and autophagic thresholds, consistent with its established anti-apoptotic roles. The dual role of Bcl-2 proteins in regulating apoptosis and autophagy [38] suggests that HAX-1 may also employ a common molecular mechanism to coordinate these prosurvival effects.

In cardiac cells, autophagy is an essential process enabling preservation of cellular homeostasis under baseline physiological conditions and adaptation to stressors such as hypoxia, nutrient deprivation and oxidative stress [19,39]. Accordingly, autophagic alterations have been reported in various cardiac disorders, further signifying the critical impact of autophagy in cardiac physiology [17,18,19,20,39]. Selective autophagy of organelles including mitochondria (mitophagy) and ER (ER-phagy) has emerged as a critical mechanism of cardiac function regulation in both health and disease [16,40]. Given the dual localization of HAX-1 at mitochondria and SR, its interaction with LC3 and its impact on autophagic flux, it is plausible that HAX-1 may participate in autophagy-related turnover of proteins associated with these compartments. 

The potential physiological relevance of this mechanism is illustrated by PLN, a key inhibitor of SERCA2a and regulator of SR Ca^2+^ cycling. Previous work has shown that PLN protein levels can be modulated by autophagy and that this is associated with changes in SR Ca^2+^ levels in cardiomyocytes [33]. Our finding that HAX-1 promotes CQ-sensitive reduction in PLN abundance (Figure 7) therefore suggests a possible link between HAX-1-mediated autophagy, PLN proteostasis and SR Ca^2+^ regulation. This interpretation is further supported by the disease-associated PLN-R14del variant, for which HAX-1 failed to promote protein reduction. Since PLN-R14del has been associated with impaired autophagosome–lysosome fusion and protein aggregation [23] and clustered perinuclear SR/ER material has been reported in cardiac tissue from PLN-Arg14del patients and mouse models [41], these observations suggest that HAX-1-dependent regulation of PLN abundance may require an intact autophagic/lysosomal pathway. 

In cardiac muscle, HAX-1 promotes cardioprotection through a multitude of different regulatory mechanisms that are specifically focused on protecting mitochondria and ER/SR compartments. In particular, HAX-1 preserves mitochondrial membrane potential and integrity of the mitochondrial transition pore through downregulation of cyclophilin-D to attenuate oxidative stress and cell death [42]. In addition, it protects the ER/SR by reducing ER stress signaling and inhibiting induction of cell death [11]. This protective effect is manifested particularly under conditions such as ischemia/reperfusion injury, where HAX-1 was shown to inhibit SERCA2 oxidation and degradation in order to preserve cell survival [43]. Under stress conditions, such as oxidative stress, a decrease in PLN levels was previously observed [44]; however, the putative role of HAX-1 in this setting remains to be explored. Stress-induced autophagy activation is a response mechanism that counteracts pathogenic processes, maintains cellular homeostasis and promotes survival [18,39]. Under such conditions, PLN degradation by HAX-1 may constitute an effective means of clearance of potentially damaged or misfolded protein towards prevention of cellular misfunction. It may therefore be hypothesized that regulation of PLN proteostasis may represent a pro-survival mechanism contributing towards alleviating Ca^2+^ mishandling under stress conditions. 

While the current study identifies a LC3/LIR-dependent mechanism contributing to the autophagy-regulatory function of HAX-1, several limitations should be acknowledged. First, the present data do not establish whether HAX-1 binds LC3 directly, nor do they define the individual contribution of each predicted LIR motif. Second, the findings rely largely on overexpression and knockdown approaches in HEK293 cells and differentiated H9c2 cardiac myotubes, which may not fully reflect endogenous protein stoichiometry or mature cardiomyocyte physiology. Third, although the PLN and PLN-R14del data support a possible connection between HAX-1-mediated autophagy and SR protein proteostasis, they do not establish functional relevance in cardiac disease in vivo. Further studies in primary cardiomyocytes, cardiac tissue and in vivo stress models, including ischemia/reperfusion injury, will therefore be required to define the physiological and disease relevance of this mechanism. 

## 5. Conclusions

The present study identifies a previously unrecognized LC3/LIR-dependent association by which HAX-1 modulates autophagic activity. HAX-1 associates with LC3 and p62, and deletion of its N-terminal LIR-containing region reduces LC3 association, decreases autophagic flux and impairs autophagy-dependent clearance of HAX-1 itself. In cell-based models, HAX-1 also promotes lysosome-dependent reduction in its SR binding partner PLN. These findings expand the mechanistic basis of HAX-1 activity and suggest that HAX-1-mediated autophagy may contribute to SR protein proteostasis, a possibility that requires further validation in primary cardiomyocytes and in vivo cardiac disease models. 

## Data Availability

The original contributions presented in this study are included in the article and Supplementary Material. Further inquiries can be directed to the corresponding author.

## Supplementary Materials

The following supporting information can be downloaded at https://www.mdpi.com/article/10.3390/cells15161459/s1: Figure S1: Representative images of undifferentiated (Day 0) and differentiated (Day 7) H9c2 cells. Scale bar 100 μm; Figure S2: HAX-1 overexpression enhances autophagic flux, as evident by (**A**) immunofluorescence and (**B**) quantification analysis of LC3 puncta in GFP- or GFP-HAX-1-transfected H9c2 cells treated with CQ (n = 80–90 cells from 3 independent experiments; *** *p* < 0.0001, one-way ANOVA). Scale bar 10 μm; Figure S3: HAX-1-mediated autophagic degradation of PLN in differentiated H9c2 cells. (**A**) Representative immunoblot analysis of GFP-PLN levels in H9c2 cells co-transfected with GFP-PLN and myc control, co-transfected with myc control followed by rapamycin (Rap) treatment, or co-transfected with myc-HAX-1. GAPDH was used as a loading control. (**B**) Quantification of GFP-PLN levels showed that rapamycin significantly reduced GFP-PLN abundance. Co-expression of myc-HAX-1 similarly reduced GFP-PLN levels, suggesting a role for HAX-1 in autophagic regulation of PLN proteostasis (n = 4 independent experiments; * *p* < 0.05, ** *p* < 0.001; one-way ANOVA followed by Tukey’s post hoc test); Figure S4: LIR-prediction bioinformatic analysis of Bcl-2 and BclxL anti-apoptotic proteins (https://ilir.warwick.ac.uk/index.php) (accessed 4 November 2024).

## Abbreviations

The following abbreviations are used in this manuscript:

HAX-1	HS-1-associated protein X-1
LC3	Microtubule-associated protein light chain 3
p62	Sequestosome-1
PLN	Phospholamban
SERCA2	SR Ca^2+^ transport ATPase
SR	Sarcoplasmic reticulum
Hsp90	Heat shock protein 90
GFP	Green fluorescent protein
LIR	LC3-interacting region
