# Supplementary material for: HAX-1-Mediated Autophagy Modulation Involves N-Terminal LC3-Interacting Motifs
Source: Cells. 2026 Aug 14;15(16):1459. doi: 10.3390/cells15161459 (PMC13510320; doi:10.3390/cells15161459)
Supplement: Supplementary file 1 [file cells-15-01459-s001.zip › cells-4479433-supplementary.pdf]

Day 0 Day 7

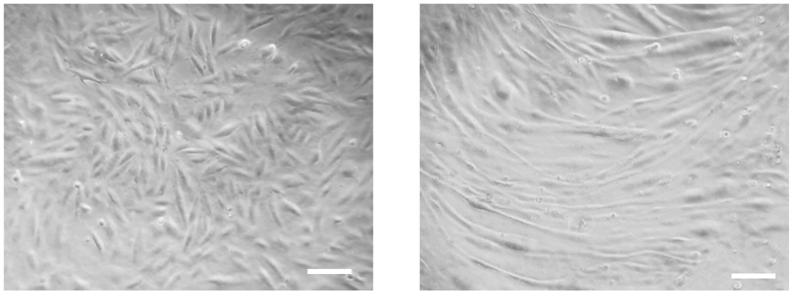

Undifferentiated Differentiated

Supplementary Figure S1

A

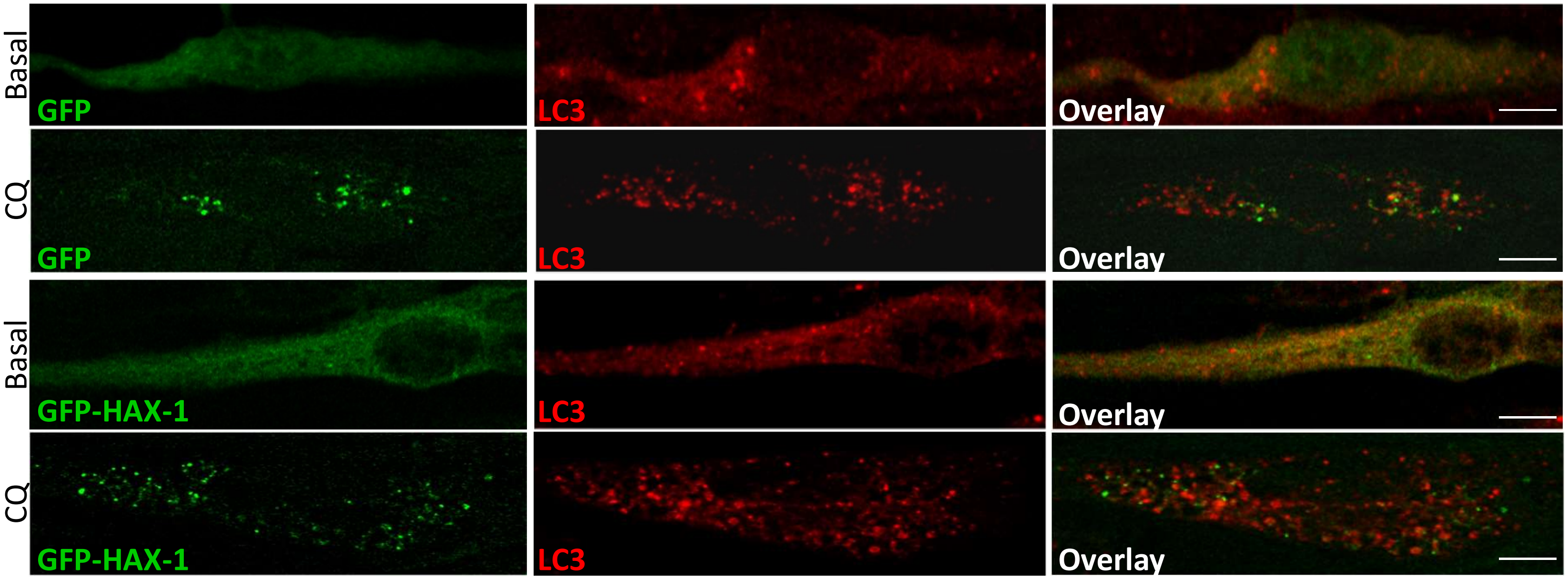

B

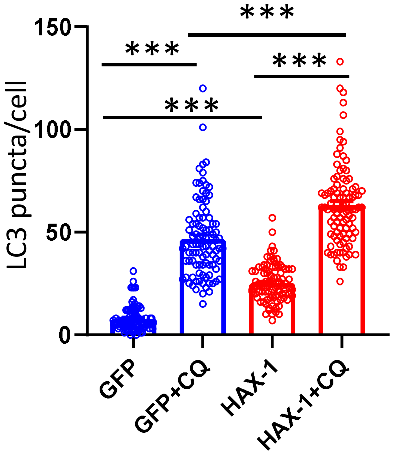

Supplementary Figure S2

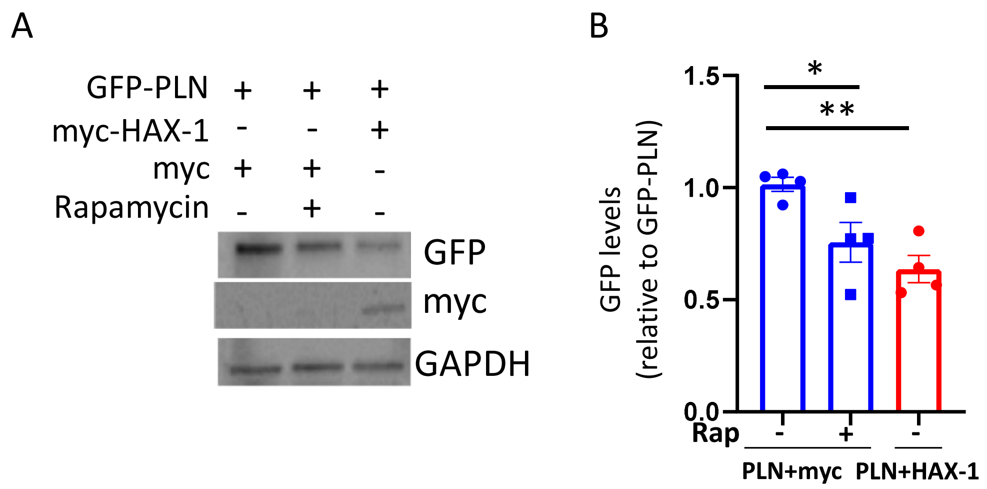

Supplementary Figure S3

Bcl2

Motif	Start	End	Pattern	PSSM Score	LIR in Anchor
xLIR	212	217	FSWLSL	18	No
WxxL	128	133	GRFATV	4	No
WxxL	142	147	VNWGRI	9	No
WxxL	151	156	FEFGGV	5	No
WxxL	196	201	DAFVEL	12	No
WxxL	210	215	FDFSWL	10	No

BclxL

Motif	Start	End	Pattern	PSSM Score	LIR in Anchor
xLIR	189	194	DTFVEL	15	No
WxxL	25	30	SQFSDV	6	Yes
WxxL	103	108	RAFSDL	6	No
WxxL	121	126	QSFEQV	8	No
WxxL	135	140	VNWGRI	9	No

Supplementary Figure S4
